# Evolution with Stochastic Fitness and Stochastic Migration

**DOI:** 10.1371/journal.pone.0007130

**Published:** 2009-10-09

**Authors:** Sean H. Rice, Anthony Papadopoulos

**Affiliations:** 1 Department of Biological Sciences, Texas Tech University, Lubbock, Texas, United States of America; 2 Department of Biological Sciences, Texas Tech University, Lubbock, Texas, United States of America; University of Bristol, United Kingdom

## Abstract

**Background:**

Migration between local populations plays an important role in evolution - influencing local adaptation, speciation, extinction, and the maintenance of genetic variation. Like other evolutionary mechanisms, migration is a stochastic process, involving both random and deterministic elements. Many models of evolution have incorporated migration, but these have all been based on simplifying assumptions, such as low migration rate, weak selection, or large population size. We thus have no truly general and exact mathematical description of evolution that incorporates migration.

**Methodology/Principal Findings:**

We derive an exact equation for directional evolution, essentially a stochastic Price equation with migration, that encompasses all processes, both deterministic and stochastic, contributing to directional change in an open population. Using this result, we show that increasing the variance in migration rates reduces the impact of migration relative to selection. This means that models that treat migration as a single parameter tend to be biassed - overestimating the relative impact of immigration. We further show that selection and migration interact in complex ways, one result being that a strategy for which fitness is negatively correlated with migration rates (high fitness when migration is low) will tend to increase in frequency, even if it has lower mean fitness than do other strategies. Finally, we derive an equation for the effective migration rate, which allows some of the complex stochastic processes that we identify to be incorporated into models with a single migration parameter.

**Conclusions/Significance:**

As has previously been shown with selection, the role of migration in evolution is determined by the entire distributions of immigration and emigration rates, not just by the mean values. The interactions of stochastic migration with stochastic selection produce evolutionary processes that are invisible to deterministic evolutionary theory.

## Introduction

Migration is both an ecological and an evolutionary process. Recognition of its importance in evolution goes back at least to Wright [1] and Haldane [2]. Because the pattern of migration influences the very structure of a population, it plays an important role in theories of speciation [3]–[5], extinction [6], kin selection [7]–[9], and the consequences of multilevel selection [10].

Any truly general theory of evolution must thus include migration. Unfortunately, migration presents some substantial challenges to modelers. First, unlike mutation, which can reasonably be treated as a weak process, migration rates in nature can be very high. This limits the applicability of certain mathematical methods, such as diffusion theory, that require that directional forces be weak.

Second, migration involves two very different processes: immigration and emigration. There is no single, biologically obvious, way in which these two processes will be related to one another; immigration and emigration may be positively correlated, negatively correlated, or independent [11], depending on environmental circumstances.

The standard way to deal with such complications in modeling evolution, as in most of science, is to make simplifying assumptions - such as that migration is symmetrical (immigration and emigration balance one another), that migration rates are low, or that migration is a deterministic (rather than a stochastic) process. Relaxing some assumptions generally requires making others. For example, Kawecki and Holt [12] model asymmetric migration, but use a deterministic model to achieve analytical tractability. Kirkpatrick and Barton [13] and Lundy and Possingham [14] develop stochastic models, but must assume that migration rates are low.

Given the complexity of evolution with migration, some authors have turned to simulation models [15]–[17]. These have the advantage of allowing for stochasticity combined with strong directional forces, and have been used with some success to study the factors influencing species range and the maintenance of variation. Simulations, however, generally require other simplifying assumptions, such as specification of the form of the distribution of fitness values (*e.g.* poisson, normal, etc.) and the exact form of the function mapping phenotype to fitness (often assumed to be gaussian). We refer to these as “simplifying” assumptions because their purpose is to simplify the mathematics - nobody thinks that they are exactly true in most cases.

Though all scientific theories start with assumptions, some require no *simplifying* assumptions. The Price equation [18] and some of its variants [19]–[22], are based only on assumptions that we believe to be true, such as that organisms live in populations, have measurable phenotypes, and produce descendants. We might call assumptions such as these “scientific axioms” to distinguish them from the simplifying assumptions encountered in model building.

The exact equations discussed above have a limitation, though - they are deterministic. Evolution is an inherently stochastic process; we can not know with certainty how many descendants an individual will leave or what they will look like until after reproduction has taken place. The Price equation and its deterministic variants are thus exact only in hindsight, after evolutionary change has occured.

Recently, a stochastic version of the Price equation was derived [23] that treats individual fitness and offspring phenotype as random variables, meaning that each individual has a distribution of possible numbers of offspring and another distribution of possible offspring phenotypes. This equation gives an exact description of the expected evolutionary change over the coming generation in a closed population. To date, though, we have no general stochastic evolutionary theory that incorporates migration while making only axiomatic assumptions.

In this paper, we present a general equation, requiring no simplifying assumptions, for directional evolutionary change in a population subject to migration. This result generalizes the stochastic Price equation [23] by introducing immigration and emigration as distinct stochastic processes. Using this result, we show that the interactions between selection, emigration, and immigration, leads to previously unrecognised evolutionary processes.

In particular, we show that the role of migration in evolution is strongly influenced by the entire distribution of immigration and emigration rates, not just the mean values. Previous authors [24], [25] have shown that the variance in immigration rates influences expected allele frequencies in models without selection. We extend this result to cases with selection, and show that increasing the symmetrical variation in immigration rates reduces the evolutionary impact of migration relative to selection. One consequence of this is that, since there will nearly always be some variation in immigration rates, classical models that treat migration as a single parameter (with zero variance) will consistently overestimate the influence of immigration relative to local selection, and thus underestimate the potential for local adaptation and speciation.

We also show that there is an evolutionary force acting to increase the frequency, within a local population, of those strategies for which fitness is negatively correlated with migration rate. This means that a strategy may increase in frequency within a local population even if it confers a lower expected fitness than do other strategies, if it causes individuals to distribute reproduction so as to produce the most offspring when there are few immigrants.

These evolutionary processes are invisible to most classical models of evolution, which treat migration as a parameter rather than as a random variable. In order to incorporate some of the stochastic processes that we describe into classical models, we derive an “effective migration rate” that extends those used previously [25] and that allows the distribution of immigration and emigration rates to be incorporated into models with a single migration parameter.

## Results

We consider an open subpopulation, or deme. All individuals currently in the deme are referred to as residents, and those that arrive from outside the deme in the subsequent interval are referred to as immigrants. An “individual” may be any biological unit that has some measurable phenotype and can leave descendants - including organisms, mated pairs, haplotypes, alleles, etc. The fitness of an individual, designated by 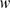, is the number of descendants that it has after a chosen interval. We use the term “descendant” broadly, to include the individual at a future time, its offspring, grand-offspring, etc. Including an individual as one of it's own descendants allows us to apply our results to cases of overlapping generations [20]. In general, descendants need not be the same type of biological unit as their ancestor; we need only be able to measure the same phenotype in both. When ancestors and descendants are of different types, it is often useful to define the phenotype as an average value. If, for example, ancestors are mated pairs of organisms, descendants are individual organisms, and we wish to study body size, then we define the phenotype as average body size, which is just the midparent value (as used in quantitative genetics) in the ancestors and individual size in the descendants. The time interval that we choose to look over is arbitrary. In many cases, it will be convenient to look over a single generation, but we could in principle choose a shorter or longer interval. Individual fitness, descendant phenotype, number of immigrants, immigrant phenotype, and whether or not an individual emigrates, are all treated as random variables, not as parameters [23]. Table 1 lists the notation used in this paper.

**Table 1:** Symbols and Notation.

Symbol	Meaning
*N*	Population size
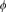	Phenotype of an individual
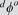	Mean phenotype of an individual's non-emigrating offspring
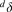	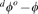
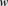	Fitness of an individual
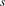	Emigration variable. 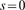 if an individual emigrates and 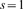 if it stays
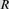	Per capita deme growth rate
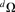	Contribution of individual resident to per capita deme growth
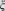	Number of immigrants, and their descendants, divided by deme size
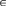	Number of emigrants divided by deme size
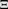	Contribution of immigrants to per capita deme growth
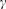	Difference between mean immigrant phenotype and mean resident phenotype
H( 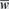 )	Harmonic mean of 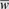
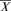 or Ave( 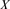 )	Average value of 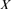 across some set of individuals
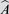 or E( 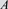 )	Expected value of random variable 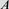
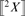	Frequency variance in the value of 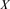 across some set of individuals
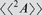	Probability variance in random variable 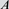
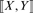	Frequency covariance, over a set of objects, between the values of 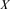 and 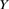
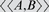	Probability covariance between random variables 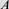 and 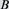

Throughout this paper, we use the term “migration” to refer to any movement of individuals into or out of a specified population. When the units that we are following are individual organisms, then this use of “migration” is synonymous with “dispersal”, as used in many ecological models. When the units that we are following are allele copies, then “migration” is synonymous with “gene flow”, as used in population genetic models.

Immigration is captured by the random variable 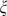, which measures the number of immigrants arriving during the chosen time interval, along with their non-emigrating descendants, divided by the current deme size (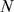). (We could as well simply define 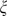 in terms of the number of descendants of immigrants, since we count an individual as one of its own descendants.) Emigration is captured by the random variable 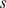. For a given descendant, 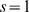 if that descendant stays in the deme and 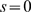 if it emigrates. For individual 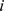, 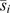 is the average value of 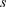 among 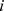
's descendants. The total number of descendants that an individual has after the specified time is its fitness, denoted 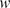, so the total number of descendants that stay in the deme is 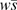. Per capita deme growth is denoted 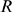, and is given by:
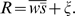
(1)


In an open deme, where there is both emigration and immigration, the contribution of resident 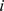 to deme growth is denoted 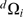, and is defined as
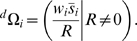
(2)


Similarly, the contribution of immigrants to deme growth, denoted 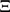, is given by:
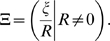
(3)


Since all future members of the population must be descended either from current members or immigrants, it must be the case that:
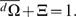
(4)


We can confirm Equation 4 simply by adding the average of Equation 2 to Equation 3.

For reasons discussed below, we need only refer to the mean phenotype of immigrants, which we denote 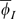. The difference between the mean phenotype of immigrants and that of residents is represented by 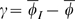.

We denote the mean phenotype of individual 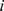
's descendants as 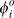. Not all of these descendants will remain in the deme, though, so what we are interested in is 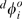 - the mean phenotype of 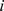
's *non emigrating* descendants. The phenotypic difference between these non emigrating descendants and their ancestor, individual 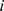, is denoted 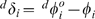.

With the exception of 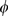 and 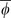, the terms defined above are all random variables, they will thus each have an associated distribution, and we will be dealing with the means, variances, and covariances of these distributions. Because we are dealing with evolution, though, we will also have to refer to the means, variances, and covariances of values within the population of organisms that we are studying. In the following discussion, it is essential to distinguish between operations over individuals in the deme (or some other finite group of objects) and operations over random variables. To make this distinction clear, we will use 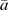 to represent the average value of 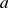 among a group of individuals, and 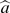 to represent the expected value of random variable 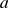. Similarly, 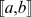 will represent the covariance between 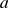 and 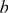 within a population, and 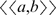 will denote the covariance between random variables 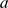 and 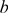 across all possible outcomes. We will refer to operations over individuals in a population as “frequency” operations. For example, the covariance term in the Price equation (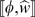 in our notation) is the “frequency covariance” between phenotype and expected fitness. Operations over random variables will be referred to as “probability” operations. We discuss this distinction in more detail in the Methods section.

### The general equation

Using the notation described above, the expected change in mean phenotype within a deme, over some (arbitrary) time interval, is given by (see Methods for derivation):
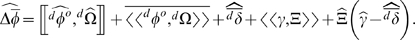
(5)


We can rewrite Equation 5 in terms of individual phenotypes (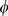) by noting that 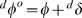. This yields:
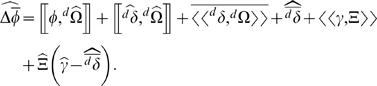
(6)


As noted, we may choose any time interval over which to calculate 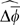. Our choice of this interval, though, will influence the values of 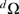 and 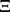, since these measure the contributions of individual resident reproduction and immigration to deme growth over the chosen interval.

(The fact that we are using single terms for immigration rate and mean immigrant phenotype does not preclude the possibility that immigrants could come from different places, since 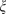 and 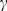 are calculated by summing over all sources of immigrants. There may be cases, though, where it would be biologically informative to distinguish between immigrants from different sources - such as when we wish to distinguish between individuals coming from nearby patches and those from distant populations. The subsection “Distinguishing between multiple immigrant populations”, in the “Methods” section, briefly explains how to modify Equation 5 to explicitly distinguish different immigrant populations.)

The different terms in Equation 6 correspond to different sets of evolutionary processes that influence directional evolutionary change. Below, we briefly describe each of these terms before focusing in detail on a few of them in the Discussion.


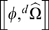
 - This term contains selection, as well as directional stochastic effects [23]. However, because 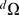 is a function of both emigration and immigration, as well as fitness, this term also captures the evolutionary consequences of the relationship between an individual's phenotype and the probability that it or its offspring will emigrate. We will expand and discuss this term in a later section.


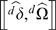
 - The covariance, across individuals in the deme, between the expected contribution to deme growth and the phenotypic difference between non-emigrating descendants and their parents. This term will be non zero if, for example, those individuals who are expected to make the greatest contribution to deme growth (highest 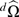) are also the ones who's offspring are expected to differ most from themselves (high 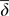). This also captures cases in which the individuals with higher than average 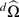 tend to have offspring for whom the probability of staying in the deme is most strongly related to their (the offspring's) phenotype.


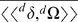
 - Average across the deme of the covariance, within an individual, between the phenotype of that individual's non-emigrating descendants (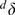) and that individual's contribution to deme growth (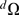). This term will be non-zero when, for example, the number of descendants that an individual produces has a direct effect on the phenotype of those descendants (as in cases of a tradeoff between clutch size and offspring size [26]), or on their probability of emigrating.


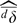
 - The expected average phenotypic difference between those descendants that stay in the deme and the phenotype of their parents. This includes any processes that cause offspring to differ from their parents (*e.g.* mutation, recombination, etc), as well as any uniform effect of phenotype on emigration (such as when the larger offspring are more likely to emigrate, regardless of the parents' phenotype).


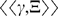
 - Covariance, across all possible outcomes, between mean immigrant phenotype and the contribution of immigrants to deme growth. This will be non-zero when, for example, immigrants are expected to differ most from natives (high 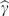) at those times when the immigration rate is high.


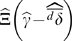
 - The expected contribution of immigrants to deme growth multiplied by the expected difference between immigrant phenotype and the phenotype of natives that do not emigrate. We will discuss the consequences of this term in a later section.

Each of these terms contains much more biology than is apparent at first glance. This is because 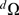 and 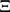 are ratios of correlated random variables, the expectations of these thus contain all of the joint moments of those variables. These moments can be examined by expanding the main terms.

The term 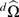 is the expected contribution of an individual resident to deme growth. In order to see what biology underlies this term, we can expand it (see Methods for the general equation) to get:
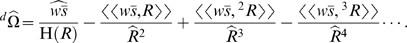
(7)


Here, 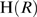 is the harmonic mean of 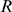, and 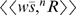 is the 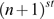 mixed central moment defined by 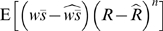. We can expand 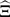 in a similar way:
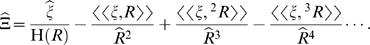
(8)


Here, it is useful to define a term to capture the overall emigration rate. We thus define 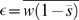 as the average per capita rate of emigration from the deme. We can now write 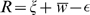. Using this fact to expand the second term on the righthand side of Equation 8, we can write 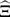 (writing only out to the second order terms) as:
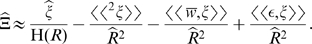
(9)


Equation 9 shows that the evolutionary impacts of immigration are influenced not only by the mean immigration rate (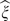), but also by the variance in 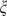 as well as by how immigration rate covaries with within deme reproduction (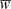) and with emigration (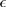). Equation 9 includes only second order moments (variances and covariances) because it represents only the first two terms in Equation 8. The subsequent terms in Equation 8 contain the higher moments of 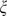 as well as the nonlinear relations between immigration, emigration, and within deme reproduction (captured by the higher mixed moments). In subsequent sections, we will discuss some of the evolutionary consequences of the relations shown in Equation 9.

## Discussion

Equations 5 and 6, which are equivalent, encompass all factors, both deterministic and stochastic, that contribute to change in mean phenotype in an open population (one subject to immigration or emigration). If migration is eliminated, then Equation 5 becomes Equation 1 in Rice 2008. These equations assume only a population of things, with measurable phenotypes, that leave descendants. Equations 5 and 6 are thus essentially two versions of a stochastic Price equation with migration. The “individuals” in the local population may be alleles, haplotypes, organisms, groups, or any other biological unit to which we can assign a phenotype and identify descendants. As with the Price equation, ancestors and descendants need not be the same kind of biological unit, so, for example, ancestors may be diploid individuals and descendants may be haplotypes. Furthermore, an individual can count itself at a later time as a descendant, allowing for overlapping generations.

In a closed population, 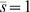 (no emigration), 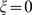 (no immigration), so 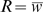 and 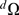 becomes simply 
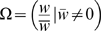
 [23]. A number of authors have identified the expected value of this term, 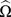 or “expected relative fitness”, as playing an important role in directional evolution [23], [27], [28]. Equation 6 shows that in an open population (where there is a possibility of emigration or immigration), 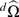 plays the equivalent role. In other words, what matters is not an individual's fitness relative to the average fitness in the deme, but rather the number of descendants of that individual who remain in the deme (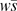) relative to the per capita growth rate of the deme (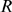), which includes immigrants. (Note that “per capita” here refers simply to the value divided by the initial deme size). If 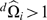, then the descendants of individual 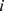 are expected to comprise an increasing proportion of the deme. All probabilities are calculated conditional on 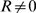, which is equivalent to saying that the local population does not go extinct. This condition is important because 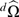 and 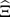 are undefined if 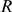 can equal zero. Conditioning on 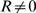 also makes biological sense, since 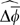 *should* be undefined when the population goes extinct. Note that in most natural populations, the probability of a local population going extinct in a given generation is sufficiently low that conditioning on 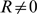 does not appreciably change the calculations.

In a closed population, the average relative fitness must be one (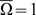), since all individuals in the future are descended from current members of the population. This is not the case in an open population, since some future population members are immigrants. We thus have a second term, 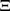, that measures the contribution of immigrants to deme growth (Equation 3).

Equations 7 and 9 show that, potentially, all of the moments of the distributions of fitness, immigration, and emigration, contribute to evolutionary change. Below, we discuss a few of the consequences of these moments for evolution. First, we give a brief intuitive explanation as to why it is important to consider the entire distributions of some variables, but not others, when calculating the change in mean phenotype.

### Evolution is influenced by the entire distributions of immigration, emigration, and fitness

To illustrate why we must sometimes consider the entire distribution of some parameter just to calculate the change in mean phenotype, consider the most well studied case: fitness. A number of studies have shown that when per capita population growth rate is treated as a random variable, rather than a fixed parameter, then directional evolution is influenced not only by the expected fitness of each phenotype, but also by the variance and other moments [23], [28]–[33]. This follows from the fact that, in a closed population, change in mean phenotype is inversely proportional to mean population fitness (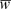). To see why this matters, consider a trait, 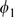, that confers high variance in fitness on individuals who express it. The fitness of 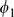 individuals tends to covary with 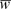 more strongly than does the fitness of individuals with lower variance in 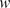. Thus, when 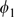 individuals are doing well, 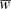 also tends to be high, so the increase in the frequency of 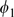 is relatively small. By contrast, when 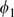 individuals happen to be doing poorly, 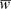 tends to be small, so the decrease in the frequency of 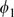 is relatively large. The frequency of 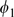 thus takes larger steps when decreasing than when increasing. The trait 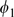 may thus be expected to decrease in frequency relative to an alternate trait, 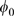, that confers a low variance in fitness, even if the expected fitness of 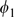 individuals is slightly higher than the expected fitness of 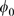 individuals. If offspring have the same phenotype as their parents (heritability = 1), then what determines which trait will increase is not expected fitness (or the geometric mean fitness [23]), but rather the expected relative fitness conditional on the population not going extinct, 
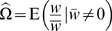
 [23], [27], [28]. (If offspring may differ from their parents, such that 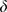 is a random variable, then 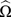 alone is insufficient to determine 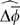 [23]).

In an open population or deme, change in mean phenotype is inversely proportional to 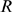, rather than to 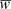. Since immigration and emigration rates contribute to 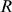 (by Equation 1) the variance and other moments of these terms now influence evolution for the same reason that variation in fitness does. For example (as elaborated below), if variation in immigration rates contributes substantially to variation in deme growth rate (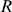), then during times of high immigration, 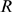 tends to be large, reducing the magnitude of change and thus reducing the impact of immigration.

Terms that measure only the phenotypes (but not numbers) of individuals, such as 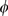, 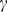, and 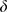, do not contribute to 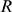. We thus need consider only the mean values of these variables in calculating the expected change in mean phenotype.

### The relation between migration and selection

The effects of selection within a deme are contained in the term 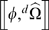. The expected contribution of individual residents to deme growth, 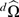, contains all of the moments of the individual's fitness distribution. Expanding 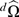 (Equation 7) shows that individual fitness (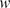) never appears by itself. Instead, 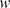 is always multiplied by 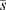, the proportion of an individual's descendants that remain in the deme. Selection is thus captured by the covariance of 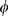 with 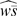. We can break up 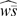 to yield:
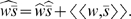
(10)


Substituting Equation 10 into Equations 1, 2, and 7, we get:
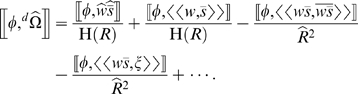
(11)


If there is no correlation between propensity to emigrate and phenotype, then increasing emigration (reducing 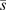) will reduce the value of 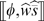. If there were no immigration, then this would not reduce the expected selection differential because 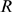 would be reduced to the same degree (Equation 7). (Random emigration might even increase the expected selection differential if the variance in 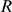 increased [23]). With immigration, though, 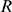 may remain large even if 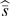 is small. In such cases, random emigration will reduce the efficacy of selection.

Any covariance between emigration and phenotype, on the other hand, essentially behaves like selection. In particular, if the offspring of parents that are poorly adapted to their local conditions (have relatively low 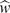) are also those most likely to emigrate (low 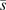) (*e.g.* small individuals are selected against and their offspring are particularly likely to emigrate), then this can amplify the value of 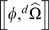, thus amplifying expected directional change. Note that this is expected if the most poorly adapted individuals (as measured by low 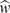) are the most likely to emigrate.

The term 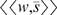 is the covariance, within an individual, between that individual's fitness and the proportion of its descendants that will remain in the deme. This term will be non-zero when, for example, producing more offspring has the direct effect of increasing the chances that any one of them will emigrate. By contrast, cases in which the proclivity to emigrate is related to expected fitness, or to parental phenotype, are captured by 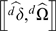.

Whenever the probability of emigration is influenced by local population density, it is likely that 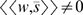. A number of studies have demonstrated density dependent emigration [34]. These include examples of both positive density dependent emigration, in which case 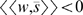 [35]–[38], and negative density dependence in emigration, in which case 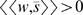 [39], [40].

A more direct influence on 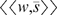 would be a causal relationship between the number of siblings that an individual has (independent of local population density) and that individual's probability of emigrating. A number of theoretical models of kin selection predict such a relationship as a consequence of competition between siblings [7], [8], [41], [42], and experimental studies have confirmed that the number of local relatives can have an effect on emigration probability, independently of population density [43]–[45].

The third term on the righthand side of Equation 11, containing 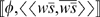, captures the tendency of populations to be pulled towards phenotypes with minimum variance in 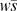. This is the equivalent, in an open population, of the “even moment effect” described in Rice [23]. The biology behind this is the same as the case for a closed population in which phenotypes differ in the variances of their fitness distributions (discussed in an earlier section).

Finally, the term 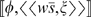 in Equation 11 shows that evolution within a deme is influenced by how distributions of 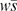 for different phenotypes covary with immigration rate. The fact that this term is negative means that, all else held equal, the phenotype for which 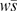 is most strongly negatively correlated with immigration rate (*i.e.* produces the most non emigrating descendants when there are few immigrants) should increase in frequency.

**Figure 1:**
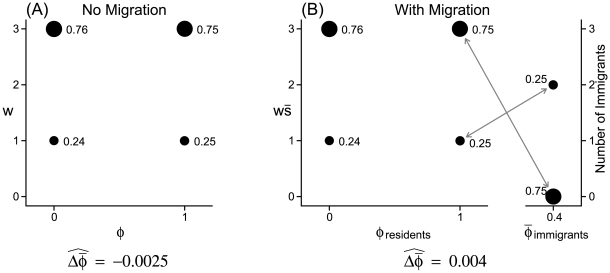
The consequences of a negative correlation between fitness and immigration rate. A) shows the fitness distributions for two phenotypes in a closed population. In the simple case of two individuals, one with 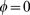 and one with 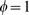 (so 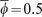), the slightly higher expected fitness of 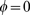 individuals causes the expected change in mean phenotype to be negative. B) shows the situation with variable immigration (and no emigration). Immigration rate is independent of the fitness of 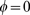 individuals, but negatively correlated with the fitness of 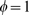 individuals. Though the mean phenotype of immigrants is lower than the mean phenotype in the deme, and selection in the deme favors a lower phenotypic value, mean phenotype is expected to increase in this case because of the fact that 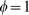 individuals do best at those times when low immigration leads to low deme growth rate. The same pattern holds in a deme of size 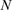 when 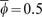, individuals with a particular phenotypic value always do well or poorly together, and the number of immigrants fluctuates between 0 and 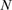.

This leads to a seemingly paradoxical prediction: In a population with weak selection favoring one phenotype over another, introducing immigrants in such a way that the rate of immigration is negatively correlated with the fitness of the less fit phenotype (and independent of the most fit phenotype) could actually cause the expected direction of evolution to reverse, towards increasing frequency of the less fit phenotype, and away from both the more fit phenotype and the mean phenotype of the immigrants. Figure 1 shows an example of a simple case in which this happens.

This is an example of what Rice [23] called a “directional stochastic effect”. It results from the fact that the phenotype with the most negative value of 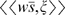 produces the most descendants at times when the potential for increase in frequency is high (since 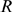 is relatively low). This effect, by itself, is unlikely to lead to fixation of the less fit phenotype (in the example in Figure 1B, the point at which 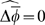 is near 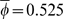). However, by shifting the distribution of strategies within a deme towards those that contribute to deme growth most when immigration is low, this process could influence population dynamics by reducing fluctuations in 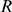.

### Variance in immigration rates

Equation 9 shows that increasing the variance in immigration rate (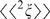) reduces the overall impact of immigration. This phenomenon has been noted previously in models of allele frequency change (Nagylaki 1979) and allelic diversity (Whitlock 1992). Equation 9 shows that this is a consequence of the impact of immigration on deme growth. When the number of immigrants entering the population is high, 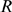 also tends to be high (by Equation 1), so overall change in mean phenotype is reduced relative to times in which the number of immigrants is relatively low. This explains why increasing the variance in immigration rate (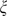) reduces the impact of immigration relative to selection within the deme; when 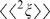 is large, immigration rate covaries strongly with 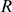.

**Figure 2:**
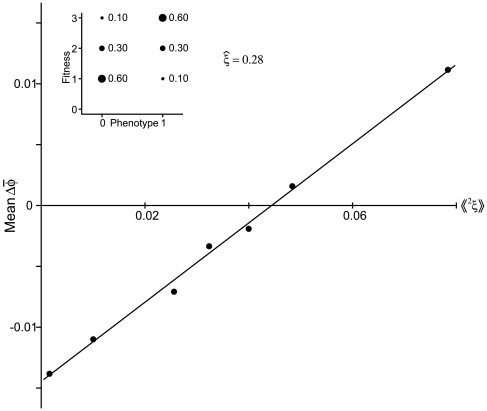
The influence of the variance in immigration rates, 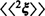, on the expected change in mean phenotype. In this example, selection favors 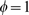 (the fitness distributions are shown in the inset figure) while immigrants have 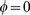. The dots in the main figure represent the means of 10,000 runs of an individual based simulation with the same expected immigration rate (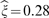) but different variances in 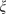. In each case, the initial deme size was 50, and emigration was independent of both immigration and phenotype.

Figure 2 shows the results of individual based simulations that illustrate the effect of 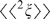 on the direction of evolution. In the example shown, selection within the deme favors individuals with a high value of 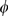, but immigrants have a low value of 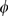. For this system, the expected change in mean phenotype is negative (migration predominates) when the variance in the immigration rate 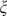 is small, but becomes positive (selection predominating) as we increase 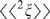. The relation is very nearly linear, as expected from Equation 9; however, the relation will ultimately be nonlinear because of the effects of higher moments of 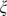, but these do not become apparent until the variance is quite large.

**Figure 3:**
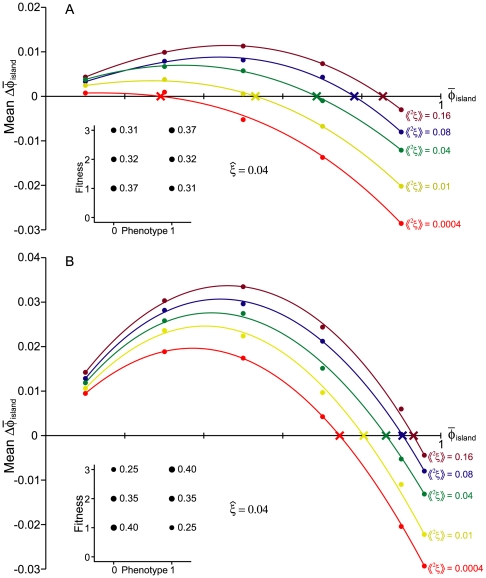
The relation between variance in immigration, 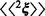, selection, and 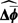 in an island-continent model. The colored lines represent 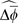 on the island as a function of current mean island phenotype for different variances in immigration rate (each colored dot represents the mean of 10,000 runs of an individual based simulation). Selection on the island favors 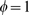 (inset figures). 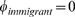, 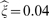, and 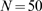. Colored “
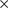
” symbols mark the values of mean island phenotype at which 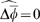. A) Relatively weak selection on the island. B) Stronger selection on the island.

The fact that the impact of migration on phenotypic change is a decreasing function of 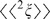 means that the distribution of migration rates has a strong effect on the potential for local adaptation. This is illustrated in Figure 3, which shows the effects of changing 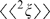 in an island-continent model. In the examples shown, selection on the island favors individuals with phenotype 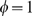, while immigrants from the continent all have 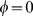. The curves in Figures 3A and 3B show the expected change in mean phenotype (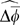) on the island as a function of the current mean phenotype on the island. Each curve is for a different value of 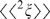, with 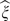 held constant. the point where the curve of 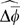 crosses zero is the expected equilibrium mean phenotype for the island.

Figure 3A shows that increasing the variance in immigration rates, keeping the expected rate constant, can have a substantial effect on the degree of local adaptation in the island population. In that example, a steady influx of two individuals per generation keeps the mean phenotype on the island quite close to that of the continent. The same average immigration rate, but resulting from irregular pulses of immigrants, allows for substantial local adaptation on the island.

In a case such as this, with two competing strategies (such as two alleles), selection becomes weak near the boundaries because of the reduction in phenotypic variance. Figure 3B shows, though, that the island can still get very close to fixation of the strategy favored there, so long as the variance in immigration is high.

### The relation between immigration and native reproduction

As the above discussion implies, the evolutionary impact of immigration is strongly influenced by the proportional contribution of immigrants to deme growth. It is thus not surprising to find the term 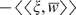 in Equation 9. If the covariance between immigration rate and native reproduction is positive, then this will reduce the evolutionary impact of immigrants, since they will tend to arrive during periods of high 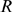. We might expect this scenario when immigration is driven by resource based habitat selection, since periods of high 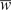 within a deme will tend to be periods of high resource availability within that deme, attracting many immigrants.

### The relation between immigration and emigration

It is important to note that immigration and emigration may show any pattern of covariation even if, over time, migration is “balanced” in the sense that the average immigration rate equals the average emigration rate. A common assumption in population genetic models is that immigration and emigration are exactly balanced within each generation, meaning that every emigrant is immediately replaced by an immigrant. This assumption has the effect of imposing a strong positive covariance between 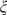 and 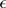. While such strict symmetry is possible, there is no biological reason to expect it to be common. We thus consider the evolutionary consequences of different possible relationships between immigration and emigration.

### No correlation between immigration and emigration

If immigration and emigration are uncorrelated, then 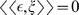. We expect this in cases in which migration is influenced by environmental or social factors that vary independently in different demes. Note that it is also appropriate to treat immigration and emigration as independent either when emigration does not happen, as when we are studying the dynamics of a sink in a source-sink model [46], [47], or when emigration is simply irrelevant to the question being asked. This last condition applies to models that focus on the probability that two randomly chosen gene copies are identical by descent [25], [47], [48].

It is likely that in many natural systems, there will be some association between immigration and emigration rates. In such cases, the nature of the association has a strong effect on the importance of migration as an evolutionary force.

### Positive correlation between immigration and emigration

If Immigration and emigration are positively correlated (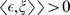), then the contribution of variable migration to variation in deme size is small, since times in which many individuals are entering the deme are also times in which many natives are leaving. This would be the case if deme size were strictly regulated, such that each individual that leaves is immediately replaced by a new immigrant. Strictly symmetric migration of this sort is assumed in many population genetics models, in which all migration is collapsed into a single parameter (often designated 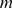) that measures the average proportion of the population replaced by migrants each generation. In such models, 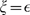, so 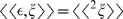, and the second and fourth terms on the righthand side of Equation 9 cancel one another out.

Though the assumption of strictly symmetric migration is usually made for the sake of mathematical simplicity, there are likely to be environmental conditions that produce a positive association between immigration and emigration, though we expect that these will usually have a correlation less than 1. One example would be a case in which individuals can migrate between local populations only at specific times, such as periods of low sea level at which previously isolated islands are connected. More generally, if the ability or inclination of individuals to migrate is influenced by an environmental factor that varies in time but, at any one time, influences all demes in the same way, then we expect that 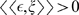.

The evolutionary consequences of a positive association between immigration and emigration will be an increase, relative to the case of 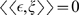, in the impact of immigration relative to selection within a deme. This follows from the fact that, if 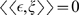, then the fourth term on the righthand side of Equation 9 is of the same sign as 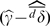, which measures the difference between the mean phenotype of immigrants and that of natives that remain in the deme. For example, if 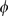 is body size, and immigrants are on average larger than natives who stay in the deme, then 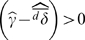 and 
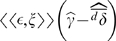
 will shift the population more towards the phenotype of immigrants.

### Negative correlation between immigration and emigration

A negative correlation between immigration and emigration (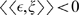) would be expected in a population of habitat selectors in which the principle reason for migration is differential patch quality. When a particular patch (corresponding to a deme) is particularly rich in resources, then individuals from other patches are expected to move in and natives are expected to stay put.

A negative relation between 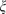 and 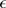 will reduce the impact of migration relative to selection. In such cases, periods of high immigration are also periods in which many natives stay in the deme, meaning that selection is particularly effective at such times as well.

### Effective migration rate

As the above discussion shows, the distributions of immigration and emigration, and their joint distributions with resident fitness, can have a strong effect on phenotypic evolution. These stochastic evolutionary processes are often not considered, though, in models of character evolution. Even the effect of variance in immigration, which has been noted previously [24], [25], is often neglected in evolutionary models involving migration (for models that do consider the variance in immigration, see [14] and [49].)

One place where stochastic migration and fitness are likely to be important is in the study of speciation. Figure 2 shows that changing the variance in immigration rates, keeping the mean constant, can substantially change the potential for local adaptation. A number of models of sympatric and parapatric speciation have shown that migration has a strong effect on the chances that complete reproductive isolation will arise between demes [3], [50]–[52], but these have treated migration as a parameter, rather than a random variable, and have not considered the joint distribution of migration and selection within a deme.

One way to introduce stochastic migration into such models, without rebuilding them from scratch, is to use an “effective” migration rate, 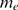. If a local population can fluctuate in size but is not expected to increase or decrease too fast (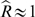), migration rates are low, and the probability of an individual emigrating is independent of its phenotype, then we can approximate the effective migration rate as:
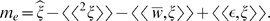
(12)


Equation 12 is an effective migration rate because it measures the evolutionary impact of migration, taking into account the impact of immigration and emigration on deme growth. If there is strict symmetrical migration within each generation (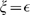 so 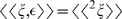), and mean fitness within the deme is independent of migration rate (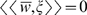), then 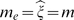.

**Figure 4:**
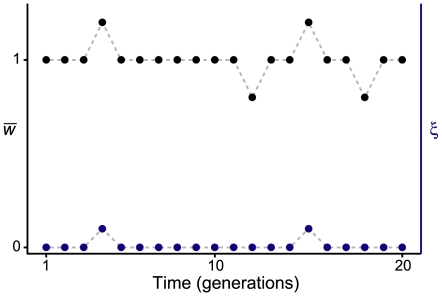
Hypothetical example of a local population in which immigration (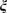) and within deme mean reproductive rate (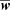) vary over time. 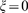
 with probability 0.9 and 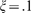 with probability 0.1, and 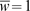 with probability 0.8, 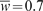 with probability 0.1, and 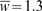 with probability 0.1. In this case, 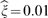 and 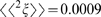. Emigration is independent of both 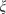 and 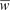. If immigration were independent of 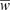 and of emigration, then the effective migration rate is 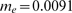. If, however, immigration and within deme reproduction are strongly correlated, so that immigrants always arrive during times of high within deme reproduction (as illustrated), then 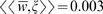, so 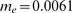.

Considering the terms on the righthand side of Equation 12, the term that has the greatest potential to significantly alter the value of 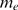 is 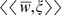; this is because, when migration rates are low (an assumption of Equation 12), the mean and variance of 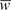 will generally be much larger than the corresponding values for 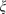 and 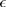. Figure 4 illustrates this point. In the example illustrated, there is substantial proportional fluctuation in immigration rates, but because the mean rate is very small, the actual value of the variance in 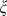 is much smaller than the mean. By contrast, though the fluctuations in within deme reproduction (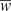) are not drastic, the fact that the mean is near 1 means that the covariance of immigration and within deme reproduction, 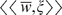, is much larger than the variance in immigration rate. In the case illustrated in Figure 4, in which times of high immigration correlate exactly with times of high within deme reproduction, the value of 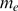 can be quite a bit less than the mean immigration rate.

Though it may be small, the variance in immigration, 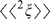, will almost never be zero, since it is quite unlikely that exactly the same number of immigrants will arrive each generation. All else held equal, we thus expect 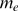 to be lower than the value of 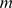 used in deterministic models unless immigration is negatively correlated with resident fitness or is positively correlated with emigration. Since the potential for parapatric speciation increases with decreasing migration [3], models with deterministic migration will thus tend to underestimate the potential for such speciation in real populations.

The effective migration rate in Equation 12 is different from the one presented by Whitlock [25] for studying 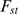. This is because, just as with effective population size, different effective migration rates will be appropriate for different questions. When calculating the probability of identity by descent for two alleles chosen from the same subpopulation [25], the rate of emigration is irrelevant. (Since they are still there, the two allele copies can not be emigrants; we are thus concerned only with the probability that they are immigrants.) By contrast, when studying phenotypic evolution within a subpopulation, both immigration and emigration matter, and thus both appear in Equation 12.

### The relation between special case models and axiomatic theories

In the Introduction, we distinguished between simplifying assumptions (postulates that we know are not strictly true but serve to make our models more tractable) and scientific axioms (postulates that we think are actually true). Most model building in biology involves either special case analytical models or simulation models. Analytical models have the advantage of providing mechanistic insight into the processes involved, but often force us to make particular simplifying assumptions for the sake of mathematical tractability, and this can render some important processes hard to model. Simulations allow us to investigate a wider range of processes, but still require many unrealistic assumptions in order to narrow the range of parameter values that need to be investigated. Furthermore, simulations tell us only what *could* happen within the range of parameter values investigated - they do not yield mechanistic insight into *why* the system behaves as it does. Though special case analytical models and simulations appear to deal differently with assumptions, in one sense they are similar: In both cases we start out by identifying the processes we think are important, then make appropriate simplifying assumptions to allow us to study these.

In building an axiomatic theory, on the other hand, we start out with what we think is actually true about the system in question, then derive from this the mathematical rules that tell us what processes are important under what circumstances. The scientific axioms from which Equations 5 and 6 follow are simply that organisms live in populations, have measurable phenotypes, leave descendants, and that we can in principle assign probability distributions to individual fitness, offspring phenotype, and immigration and emigration rates. Not surprisingly, the resulting equations have many terms, most of which can themselves be expanded to yield even more terms. In traditional model building this would be a drawback - all those terms complicating the interpretation of the model. For an axiomatic theory, though, this proliferation of terms is exactly what we want. Because we started out with only assumptions that we have good reason to think are actually true, each of the resulting terms must correspond to some real biological process - including some that we might never have thought of. For example, the importance of the correlation between within deme fitness and immigration rate (Figure 1) emerged naturally from an expansion of the terms in Equation 5 (specifically, the appearance of the term 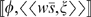). Axiomatic theories thus allow us to discover processes that were rendered invisible by the assumptions used to make special case models and simulations tractable. In many fields we have no useful axiomatic theories. When we do have them, though, they serve to clarify the fundamental relationships between different processes, and facilitate the discovery of new processes that we did not expect, but that follow necessarily from the basic facts of the system.

Finally, note that much of our analysis of Equations 5 and 6 involved considering simplified systems that isolate the effects of a particular term. We are thus still using simplifying assumptions in our analysis. Because we started out deriving an axiomatic theory, though, the simplifying assumptions come at the end, rather than at the beginning, of the analysis - after we have seen what the exact general rules look like. Axiomatic theories, when we can derive them, thus also serve as formulas for generating special case models.

## Methods

### Frequency *vs* probability operations

There are two, very different, kinds of statistical operators used in this paper: Frequency operations, denoted by straight symbols (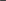 or 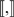), are operations over objects that have a distinct value, such as individuals in a deme or the descendants of a particular ancestor.

It is important to note that frequency operations are not the same as “sample statistics”. Though 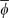 and 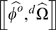 are calculated using a finite set of individuals, they are not estimates of anything else. Rather, they are the terms that actually determine the dynamics of evolutionary change within a deme [20]. This means that in calculating variances and covariances across the deme, we do not use any of the statistical corrections associated with sample statistics.

Probability operations, represented by angled symbols (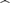 or 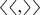), are over the possible values of random variables, such as the distribution of possible values of change in mean phenotype, or the distribution of possible fitness values of an individual.

For some values, both frequency and probability operations are relevant, though they will have very different interpretations. For example, 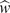 is the expected fitness of an individual, while 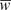 is the average fitness across the population or deme. Note that 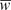 is itself a random variable, so we could calculate 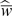, the expected average fitness, or expected per capita population growth rate. In other cases, only one kind of operation will be meaningful. For example, if 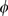 represents the current phenotype of individuals in a population, then 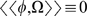. Since 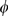 is not a random variable, it can not have a non-zero probability covariance with anything.

In order to determine the level at which a particular operation is applied, one needs to look at the objects involved. For instance, 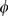 is a property of a deme because individuals within the deme each have a value of 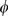, whereas 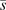 is a property of an individual ancestor, because 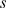 is a property of each of an individuals descendants. The average value of 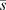 in a deme would then be 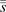.

### Rules for manipulating frequency and probability operations

Two theorems relating frequency and probability operations will be useful in the subsequent derivations. The first, demonstrated by Rice [23], says that the expected value of the average is the same as the average of the expected value:
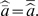
(13)


The second important relation concerns covariances and means:
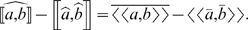
(14)


This is easily demonstrated by noting that the lefthand side of Equation 14 is equal to 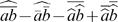, and the righthand side is equal to 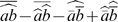. Applying Equation 13 shows that these are equal.

### Derivation of Equation 5

Consider a deme of size 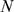. Immigrants may enter the deme such that at the end of a chosen time interval, 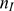 individuals are either immigrants or descendants of immigrants that arrived during the interval. The rest of the population at the end of the interval is composed of descendants of residents (individuals in the deme at the beginning of the interval). We define 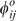 as the phenotype of the 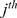 descendant of resident 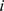 and 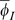 as the mean phenotype of immigrants. We can then write the expected mean phenotype in the deme at the end of the next time interval, 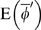, as:
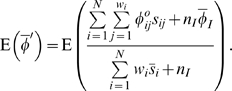
(15)


From Equation 1, and the fact that 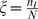, we see that the denominator of the righthand side of Equation 15 is equal to 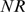. We can further simplify the first term in the numerator of Equation 15 by noting that the expected mean phenotype among those descendants of individual 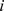 that do not emigrate, 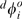 is given by:
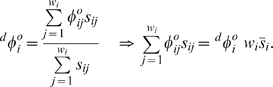
(16)


Equation 15 then becomes:
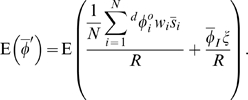
(17)


Recalling the definitions of 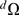 and 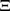 from Equations 2 and 3, and noting that 
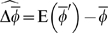
, we can write:
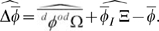
(18)


Using the fact that 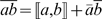 and that 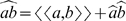, Equation 18 becomes:
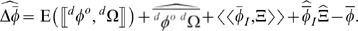
(19)


We can rewrite the first term on the righthand side of Equation 19 using Equation 14:
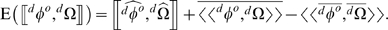
(20)


Similarly, we can use the facts that 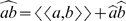 and that (from Equations 2 and 3) 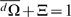 to rewrite the second term on the righthand side of Equation 19 as:
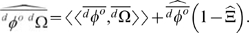
(21)


Substituting Equations 20 and 21 into Equation 19, setting 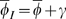, and rearranging terms, yields:
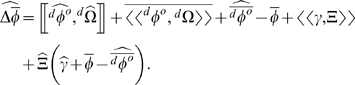
(22)


Substituting 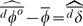 into Equation 22 yields Equation 5.

### Distinguishing between multiple immigrant populations

If we wish to formally distinguish between immigrants coming from different original populations, then each source population has it's own value of 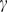, 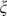, and 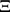. For the case of two different immigrant origins, Equations 1 and 4 become 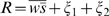 and 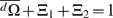. Using these facts, and following the same steps as in the derivation of Equation 5 above, we get:
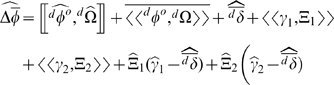
(23)


### Expanding 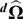 and 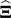


Equations 7 and 8 are obtained using the general rule for expanding ratios of random variables presented in Rice [23]. In the notation of the current paper, this is:
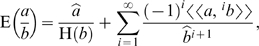
(24)where 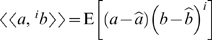.
